# From electron spin to relaxivity: a multidisciplinary perspective on first-row transition metal-based MRI probes

**DOI:** 10.1039/d5sc05827a

**Published:** 2025-10-20

**Authors:** Enrico Salvadori, Valeria Lagostina, Marco Ricci, Fabio Carniato, Mauro Botta, Carlos Platas-Iglesias, Mario Chiesa

**Affiliations:** a Department of Chemistry, University of Turin Via Giuria 9 10125 Torino Italy mario.chiesa@unito.it; b Dipartimento di Scienze e Innovazione Tecnologica, Università del Piemonte Orientale Viale T. Michel 11 15121 Alessandria Italy mauro.botta@uniupo.it; c Centro Interdisciplinar de Química e Bioloxía (CICA), Departamento de Química, Facultade de Ciencias, Universidade da Coruña 15071 A Coruña Galicia Spain carlos.platas.iglesias@udc.es

## Abstract

The electron spin is a key enabler of some of the most advanced current technologies. A prime example is the development of MRI contrast agents, where precisely engineered electron spin properties are utilized to enhance the capabilities of one of the most powerful diagnostic tools in the medical science. Clinically approved contrast agents are based on paramagnetic gadolinium(iii) complexes. However, to alleviate health and environmental concerns, as well as for specialized applications, alternatives are sought after. Due to their rich chemistry, abundance and low toxicity first-row paramagnetic transition metal ions are emerging as an appealing alternative. A large experimental effort is needed to engineer the new generation of contrast agents. The primary source of information comes from Nuclear Magnetic Relaxation Dispersion (NMRD) profiles. While fitting these profiles can, in principle, yield all the structural and dynamic parameters that influence relaxation, the underlying theoretical models demonstrate a significant challenge. The parameters affect the NMRD profiles in highly coupled, non-separable ways, meaning that a simple, unconstrained fit often results in a non-unique solution. Consequently, the independent experimental determination of some, and preferably most, of these parameters offers a considerable advantage in obtaining reliable and physically meaningful information. This perspective outlines an integrated approach that exploits Electron Paramagnetic Resonance (EPR) spectroscopy for the accurate determination of key molecular parameters. Specifically, EPR is used to quantify the rotational correlation time, the closest proton–metal distance, and the electron spin density at the proton. This methodology is particularly relevant for contrast agents based on first-row transition metal ions. We discuss the contribution of EPR in a complementary context with well-established techniques such as NMR and DFT.

## Abstract 2

EPR spectroscopy complements NMR and DFT in designing next-generation MRI contrast agents based on first-row transition metals, providing key molecular parameters for reliable interpretation of relaxometric data.

## Graphical abstract

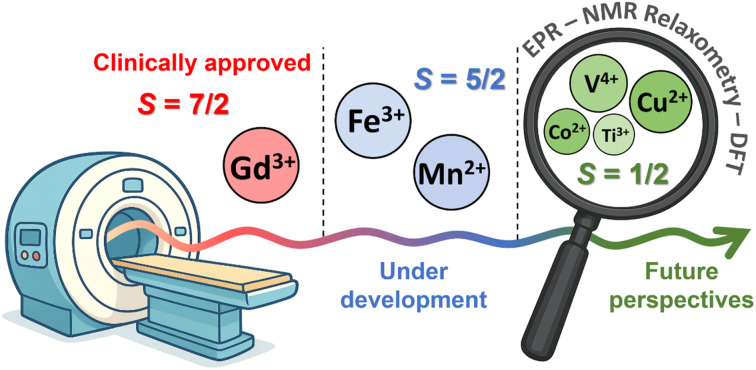

## 1. Introduction

Spins are prototypical quantum objects,^1^ readily detectable and controllable with electromagnetic radiation, and play a central role in the development of better materials for energy conversion and storage,^2,3^ more powerful and secure communication technologies,^4^ and more efficient and safer diagnostic procedures in biomedicine.^5^ Spin-bearing (paramagnetic) molecules are key in two technological areas of crucial importance for our society: Magnetic Resonance Imaging (MRI)^6,7^ and the emerging field of quantum information technologies.^8,9^ Both these applications rely on the careful engineering and control of the longitudinal and transverse relaxation times (*T*_1_ and *T*_2_).

Applications in quantum information technology require designing systems where both these parameters are long.^10^ On the other hand, contrast agents for application in MRI should efficiently shorten the relaxation times of water proton nuclei in their vicinity.^11^ These requirements are both related to the electronic and geometric structure of paramagnetic metal complexes and the nature of the coordination spheres as well as on the spin-density distribution and electron-nuclear spin interactions across subsequent ligand spheres. A detailed understanding of such factors and their dependency on the electronic and geometric structure of paramagnetic molecular complexes is therefore practically relevant, as it provides guidance for the control and implementation of sensitivity in quantum sensing, the number of operations in quantum algorithms and the efficiency of MRI contrast agents. This last aspect is the subject of this perspective.

**Fig. 1:**
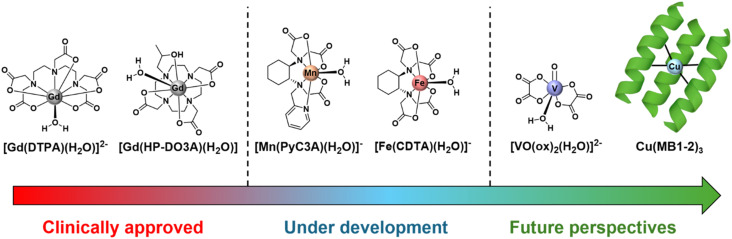
Representative chemical structures of paramagnetic metal complexes. These complexes are categorized by their current status as MRI contrast agents: Gd(iii)-based agents that are in clinical use; Mn(ii) and Fe(iii) complexes in active development; and V(iv) and Cu(ii) complexes under investigation for future perspectives.

MRI contrast agents (CAs) based on paramagnetic metal complexes have contributed to the success of MRI as a diagnostic procedure in medical science. To date, all clinically approved CAs are Gd(iii) polyaminocarboxylate chelates (Fig. 1) which are particularly effective in shortening the ^1^H relaxation times of water by virtue of their high spin state (*S* = 7/2) and long electronic relaxation times.^12^ Over tens of millions of contrast-enhanced MRI exams are performed annually around the world. It is noteworthy that, at present, 8% of the Gd market share is absorbed by the manufacture of pharmaceutical products for MRI applications. Given that Gd is an element foreign to the human body, there are increasing concerns regarding the long-term safety of Gd(iii) compounds,^13–16^ as well as the availability and supply chain of the element itself.^17^ Additionally, there is an increasing environmental concern, as anomalously high concentrations of this element of anthropogenic origin have been detected as a result of the extensive use of CAs.^18,19^ These three factors combined are prompting a revival in research efforts devoted to alternatives, with emphasis on paramagnetic first-row transition-metal (TMI) complexes.^20^ In addition to being earth-abundant and thus readily available, most TMIs are essential elements naturally present in the body. Consequently, when properly handled, they pose a reduced risk to human health, as human physiology has, within certain limits, evolved mechanisms to manage excesses of these free ions. Furthermore, a key advantage of first-row TMIs is their ability to display multiple oxidations and, consequently, spin states. This property can be exploited to engineer stimuli-responsive probes that can report on the redox landscape of the biological milieu. As paradigmatic examples, it is well known that diseases such as cancer, stroke, and atherosclerosis lead to a perturbed biological redox environment.

For these reasons, high-spin Mn(ii) and Fe(iii) complexes are currently attracting significant interest in the field (Fig. 1).^21,22^ Excellent and detailed reviews and book chapters are already available on these topics, particularly concerning Gd(iii) and Mn(ii) complexes, for which characterization methodologies are well established and yield highly reliable results.^5,20,23,24^ On the other hand, well biologically tolerated simple spin *S* = ½ systems, such as vanadyl V(iv) or Cu(ii), which are already investigated for biomedical applications (*i.e.* therapeutic agents for their antidiabetic effects, anticancer drugs or radiotracers for PET imaging) may also hold promise as potential MRI contrast agents for specific applications (Fig. 1).^25,26^ As an example, Cu(ii) and V(iv) can easily convert to their diamagnetic counterparts, Cu(i) and V(v), making them intriguing candidates as potential “smart” redox switchable sensing contrast agents. This latter field of research, however, is largely unexplored and is the main focus of this perspective.

Due to their intrinsic physical properties, TMIs will never reach the paramagnetism displayed by Gd since, for instance, the maximum number of unpaired electrons corresponds to the number of orbitals in the shell, 5 3d orbitals (*S*_max_ = 5/2) *versus* 7 4f orbitals (*S*_max_ = 7/2). However, the electron magnetic moment is just one of the relevant parameters involved. Ligand exchange rates, electronic relaxation times, metal–water distances and spin density distributions are all parameters that can be effectively tuned with ease in TMIs complexes since, as opposed to Gd, TMIs enjoy a much richer chemistry in which the nature and number of the ligands greatly influence the spin properties. This provides a limitless playground within the chemical science. In order to make these molecular entities viable alternatives to Gd, it is of central importance a thorough understanding of the spin dynamics of paramagnetic complexes of earth abundant and essential TMIs complexes, with the specific goal of designing of new and safer MRI contrast agents. In the last decade, the structural and physical factors responsible for spin dynamics of molecular *S* = 1/2 TMIs complexes have been studied in detail and reviewed, but with the intended application as potential spin qubits.^5^ Specifically, chemical structures and environments have been engineered to provide long spin–lattice and phase-memory relaxation times. While some insight can be transferred from the wealth of studies present in the literature, the application of TMIs as contrast agents presents many peculiarities that need to be addressed.

Given the large parameter space needed to model the behavior of contrast agents in this perspective, we propose and discuss a combined approach where Electron Paramagnetic Resonance (EPR) spectroscopy as well as nuclear magnetic relaxometry techniques and computational modelling provide a new and comprehensive understanding of the factors governing the spin dynamics and the relaxivity of a first row TMI including under different experimental conditions (pH, redox environment, *etc.*). This perspective highlights the unique contribution, as well as the area of applicability, of EPR spectroscopy for independently quantifying many of the crucial factors that dictate the performance of TMI-based contrast agents. We detail how these insights can be integrated with experimental relaxivity measurements and advanced quantum-chemical models to establish a robust framework for data interpretation. This approach, in turn, provides a clear set of guidelines for the rational tuning and engineering of paramagnetic TMI complexes, leveraging their fundamental electronic structure to optimize their relaxation properties.

## 2. Overview of the mechanisms of proton relaxation by paramagnetic metal ions

### 2.1 The phenomenon of paramagnetic relaxation enhancement (PRE)

Paramagnetic metal ions possess the remarkable ability to significantly augment nuclear spin relaxation rates (*R*_1_ or longitudinal and *R*_2_ or transverse) for nuclei situated in their proximity. This phenomenon, formally referred to as Paramagnetic Relaxation Enhancement (PRE), arises from the dynamic hyperfine interactions occurring between the unpaired electron spins of the metal ion and neighbouring nuclear spins. Consequently, PRE constitutes a potent mechanism for accelerating the recovery of nuclear magnetization to its equilibrium state.^27,28^

The primary driver of PRE is the electron-nucleus dipolar interaction. Unlike homonuclear or heteronuclear dipolar relaxation, which involves interactions between nuclear spins, paramagnetic relaxation is governed by the coupling between the magnetic moment of the unpaired electrons at the paramagnetic center and the magnetic moments of nearby nuclei.^29^ The strength of this interaction is proportional to the product of their respective gyromagnetic ratios (*γ*_e_ and *γ*_N_). Given that an electron's gyromagnetic ratio is approximately 660 times greater than that of a proton, the resulting dipolar interactions are significantly more potent than those involving only nuclear spins. This substantial enhancement underscores the exceptional efficiency of paramagnetic centers in promoting nuclear spin relaxation, forming the theoretical basis for their use as MRI contrast agents and paramagnetic tags in NMR spectroscopy.

### 2.2 Quantifying relaxation efficiency: relaxivity (*r*_*i*_)

The paramagnetic contribution to the nuclear spin relaxation rate of solvent molecules, denoted as *R*^p^_*i*_ (where *i* = 1 or 2), is directly proportional to the concentration of the paramagnetic species in solution.^30,31^ To facilitate meaningful comparisons between different metal ions or MRI contrast agents, it is standard practice to normalize *R*^p^_*i*_ to a fixed concentration, typically 1 mM. This normalization yields a concentration-independent metric known as relaxivity (*r*_*i*_), defined as:1
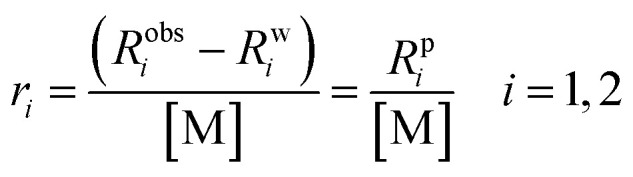
Here (eqn (1)), *R*^obs^_*i*_ is the observed relaxation rate in the presence of the paramagnetic agent, *R*^w^_*i*_ is the diamagnetic relaxation rate of the solvent (*e.g.*, water protons) in the absence of the agent, and [M] is the molar concentration of the paramagnetic metal ion. Relaxivity (*r*_*i*_) quantifies the efficiency with which a paramagnetic agent enhances relaxation per millimolar concentration unit and is expressed in units of mM^−1^ s^−1^.^32,33^ It serves as a critical figure of merit for evaluating and comparing the performance of paramagnetic systems under standardized conditions. For instance, an aqueous solution of the [Gd(DTPA)(H_2_O)]^2−^ complex at a concentration of 4.96 mM, examined at pH 7.4, 298 K, and a proton Larmor frequency of 20 MHz, exhibits an observed longitudinal relaxation rate (*R*^obs^_*i*_) of 23.90 s^−1^. Subtracting the *R*_1_^w^ of 0.38 s^−1^ (under identical conditions) and normalizing by the Gd(iii) concentration yields a longitudinal relaxivity (*r*_1_) of 4.72 mM^−1^ s^−1^.^34^

Relaxivity is not an intrinsic constant for a paramagnetic agent; it is notably dependent on the applied magnetic field strength (and thus, the Larmor frequency).^35^ Furthermore, the inherent magnetic and physicochemical properties of the specific metal ion dictate the potential range of relaxivity values. Consequently, a vast amount of relaxivity data accumulated over the past ∼30 years is typically reported under standardised conditions, often 0.5 tesla and 298 K, to allow for meaningful comparisons. Table 1 compiles longitudinal proton *r*_1_ data for selected 3d transition series and lanthanide metal ions, along with some of their key characteristics.

**Table 1:** Electronic configuration, spin state (*S*), effective magnetic moment (*μ*_eff_), longitudinal proton relaxivity (*r*_1_) and electron relaxation time (*τ*_s_) for various hydrated metal ions

	Electronic configuration	*S*	*μ*_eff_ (BM)^a^^,^^b^	*r*_1_/mM^−1^ s^−1^ (0.5 T, 298 K)^c^^,^^d^^,^^e^^,^^f^^,^^g^	*τ*_s_ (s) (1.5–3.0 T)^c^^,^^g^^,^^h^
[Ti(H_2_O)_6_]^3+^	[Ar]3d^1^	1/2	1.7	0.9	≈4 × 10^−11^
[VO(H_2_O)_5_]^2+^	[Ar]3d^1^	1/2	1.7	0.9	≈5 × 10^−10^
[Cr(H_2_O)_6_]^3+^	[Ar]3d^3^	3/2	3.9	5.6	5.4 × 10^−8^–1.7 × 10^−7^
[Mn(H_2_O)_6_]^2+^	[Ar]3d^5^	5/2	5.9	7.7	5 × 10^−8^–1.5 × 10^−7^
[Fe(H_2_O)_6_]^3+^	[Ar]3d^5^	5/2	5.9	12.1	4 × 10^−10^–1.4 × 10^−9^
[Co(H_2_O)_6_]^2+^	[Ar]3d^7^	3/2	3.9	0.1	10^−11^–10^−12^
[Ni(H_2_O)_6_]^2+^	[Ar]3d^8^	1	2.8	0.7	10^−11^–10^−12^
[Cu(H_2_O)_6_]^2+^	[Ar]3d^9^	1/2	1.7	0.9	≈3 × 10^−9^
[Gd(H_2_O)_8(9)_]^3+^	[Xe]4f^7^	7/2 (*J* = 7/2)	7.9	12.7	1.0 × 10^−9^–2.5 × 10^−9^
[Dy(H_2_O)_8_]^3+^	[Xe]4f^9^	5/2 (*J* = 15/2)	10.7^i^	0.6	0.4 × 10^−12^
[Ho(H_2_O)_8_]^3+^	[Xe]4f^10^	2 (*J* = 8)	10.6^i^	0.4	0.3 × 10^−12^

a Ref. 36

b Ref. 37.

c Ref. 38.

d Ref. 39.

e Ref. 40.

f Ref. 41 and 42.

g Ref. 43.

h Ref. 44.

i *μ*_eff_ due to total magnetic moment (*J* = *L* + *S*).

### 2.3 Fundamental relaxation mechanisms: the Solomon–Bloembergen–Morgan (SBM) framework

The theoretical basis for understanding PRE was largely established by Solomon, Bloembergen, and Morgan (SBM).^45–47^ The SBM theory and its subsequent modifications provide analytical expressions for relaxation rates, enabling the fitting of experimental data. According to this framework, the paramagnetic contribution to the relaxation rate of a nucleus influenced by a paramagnetic center arises primarily from two hyperfine interaction mechanisms:^48^

(a) Dipole–dipole (DD) interaction: this contribution arises from the long-range magnetic dipole–dipole coupling between the magnetic moment of the unpaired electron and the nuclear spin. This is a through-space interaction that is exquisitely sensitive to distance, and, for fluctuating dipoles such as in fluid solutions, decays rapidly with the inverse sixth power of the separation between the metal center and the nucleus (*r*_Me–H_^−6^). This mechanism is typically the dominant contributor to relaxivity in many biologically and chemically relevant complexes, including those based on Fe(iii), Mn(ii), Gd(iii), and other lanthanides.

(b) Scalar or Fermi contact (SC) interaction: this is a through-bond interaction resulting from the delocalization of unpaired electron spin density onto the observed nucleus *via* covalent bonds. The contact contribution is usually negligible for lanthanide complexes such as Gd(iii) due to the shielding of 4f electrons by outer shell electrons^49,50^ but can be significant for certain transition metal complexes with substantial metal–ligand orbital overlap. While often minor in the context of bulk relaxivity, contact shifts are critical in paramagnetic NMR studies of metalloproteins and small paramagnetic ligands.

A third mechanism, Curie Spin relaxation (CS), also contributes, particularly at high magnetic fields and for systems with slow molecular tumbling or short electronic relaxation times. It arises from the static magnetic field generated by the thermally averaged electron spin magnetic moment. The CS mechanism gains importance under conditions where the dipolar coupling is effectively controlled by the electronic relaxation time; this occurs when *τ*_s_ is significantly shorter than the rotational correlation time. In essence, the contribution of the CS mechanism is intrinsically linked to the chemical and physical characteristics of the metal ion itself.

### 2.4 Contributions from different water environments: the hydration sphere model

**Fig. 2:**
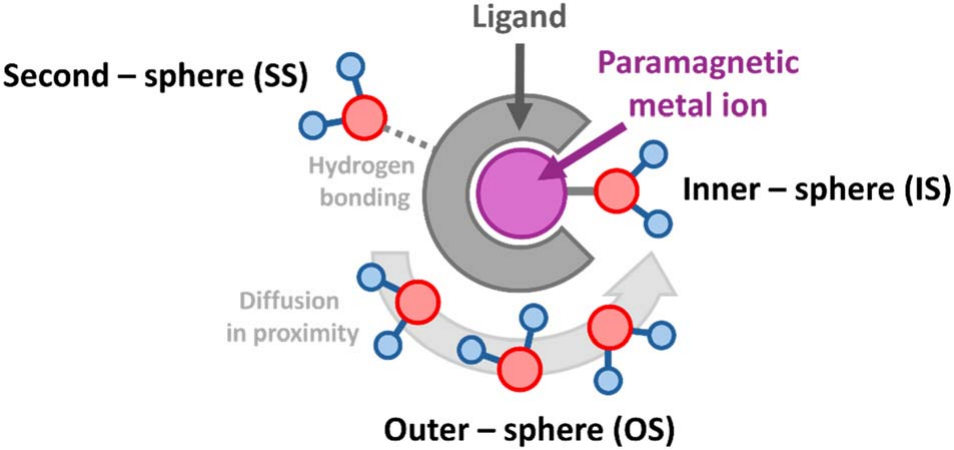
Three distinct mechanisms of water interaction with the paramagnetic metal center.

NMR relaxometry is a powerful technique for probing the dynamic interactions between water molecules and paramagnetic complexes. By measuring the relaxation rates of specific nuclear spins in water, namely ^1^H and ^17^O, we can gain detailed insights into the different environments and exchange pathways of water in the vicinity of these metal ions. This is vital for characterizing MRI contrast agents, where water relaxation efficiency is paramount. The overall observed relaxivity can be dissected into contributions from water molecules in different environments relative to the paramagnetic centre (Fig. 2):

(1) Inner sphere (IS) contribution: arises from water molecules directly coordinated to the metal ion. This is often the most significant contribution due to the close proximity and strong dipolar interaction, coupled with their exchange with bulk water.

(2) Second sphere (SS) contribution: involves water molecules not directly coordinated but transiently associated with the complex, typically *via* hydrogen bonds to the ligand. These water molecules experience a weaker, but still significant, interaction with the paramagnetic centre. Their contribution depends on their population, residence time in this sphere, and exchange rate with bulk water. For certain contrast agents, particularly those with specific ligand functionalities (*e.g.*, hydroxyl or amide groups), these “long-lived” second-sphere water molecules can substantially enhance overall relaxivity.

(3) Outer sphere (OS) contribution: accounts for water molecules diffusing freely in the bulk solution that transiently approach the paramagnetic complex. The dipolar interaction is weaker and time-averaged due to rapid translational diffusion. This contribution is generally less efficient than IS relaxation but is always present and can be more prominent for smaller complexes or those with slow inner-sphere water exchange.

The total relaxivity is thus the sum of these components (eqn (2)):

2 *r*_*i*_ = *r*^IS^_*i*_ + *r*^SS^_*i*_ + *r*^OS^_*i*_ *i* = 1, 2

Each component contributes differently based on spatial proximity, interaction strength, and dynamic behaviour. Details on the inner and outer-sphere mechanisms can be found as SI.

### 2.5 Key factors influencing relaxivity

**Fig. 3:**
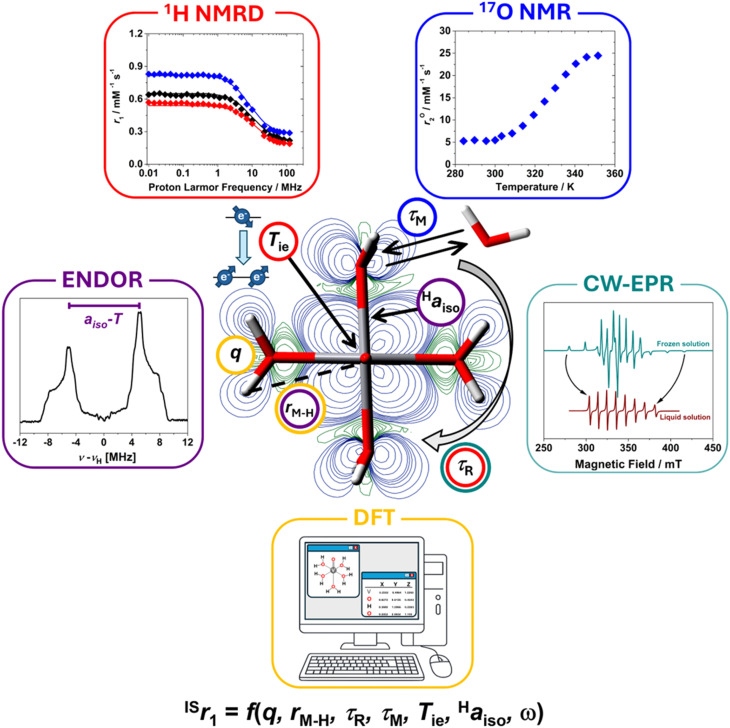
The figure illustrates the molecular and dynamic characteristics of a paramagnetic metal chelate obtained through various experimental and theoretical methods. Each parameter is color-coded to represent the specific technique used. A general function that correlates ^IS^*r*_1_ to the molecular and dynamic parameters of the probe and the magnetic field frequency (*ω*) is reported below. A more detailed explanation of this equation is provided in the SI.

The data in Table 1 illustrate that *r*_1_ is not simply proportional to the effective magnetic moment. Instead, relaxivity is a complex function of several interconnected parameters,^23^ which are graphically illustrated in Fig. 3:

• Number of coordinated water molecules (*q*): inner-sphere water molecules, directly bound to the metal ion, experience the most intense interaction with the unpaired electron spins and are crucial determinants of relaxivity.

• Water exchange rate (*k*_ex_): the rate at which inner-sphere water molecules exchange with bulk water (*k*_ex_ = 1/*τ*_M_, where *τ*_M_ is the residence lifetime). An optimal exchange rate is vital for effectively transferring the relaxation enhancement from coordinated water to the bulk solvent.

• Rotational correlation time (*τ*_R_): this parameter reflects the reorientational tumbling rate of the paramagnetic complex in solution. Dipolar relaxation efficiency is often maximized when *τ*_R_ is comparable to the inverse of the proton Larmor frequency. Larger complexes generally have longer *τ*_R_, potentially leading to higher relaxivity, especially at higher magnetic fields.

• Electronic relaxation times (*T*_1e_, *T*_2e_): These describe the relaxation rates of the metal ion's electron spins. For efficient paramagnetic relaxation, *T*_1e_ and *T*_2e_ should be sufficiently long to maintain a defined electron spin state during the interaction with water protons. The significant disparity in *r*_1_ values between Gd^III^ (long *T*_1e_) and Dy^III^/Ho^III^ (short *T*_1e_) in Table 1 highlights this factor.

• Distance between metal ion and water protons (*r*_Me–H_): The dipolar interaction's *r*_Me–H_^−6^ dependence means that even small changes in this distance, particularly for inner-sphere water, significantly impact relaxivity.

• The isotropic hyperfine coupling interaction (^H^*a*_iso_) responsible for the contact contribution, which may be significant for transition metal complexes (*i.e.* VO^2+^ and Mn^2+^) as compared to lanthanides.^25,51^ The ^H^*a*_iso_ values are generally expressed in MHz in EPR studies and in rad s^−1^ in relaxometric studies, being denoted as *A*_H_/*ħ*, with *A*_H_/*ħ* = 2π × ^H^*a*_iso_.

These parameters are, in turn, influenced by various structural and chemical attributes of the complex, such as ligand donor atoms, coordination geometry and symmetry, steric interactions, net charge, and molecular size. Consequently, tailoring a complex for optimal relaxivity at a specific frequency is a significant challenge in coordination chemistry, requiring rational ligand design to balance these factors while maintaining thermodynamic stability and/or kinetic inertness.

### 2.6 Magnetic field dependence and NMRD profiles

**Fig. 4:**
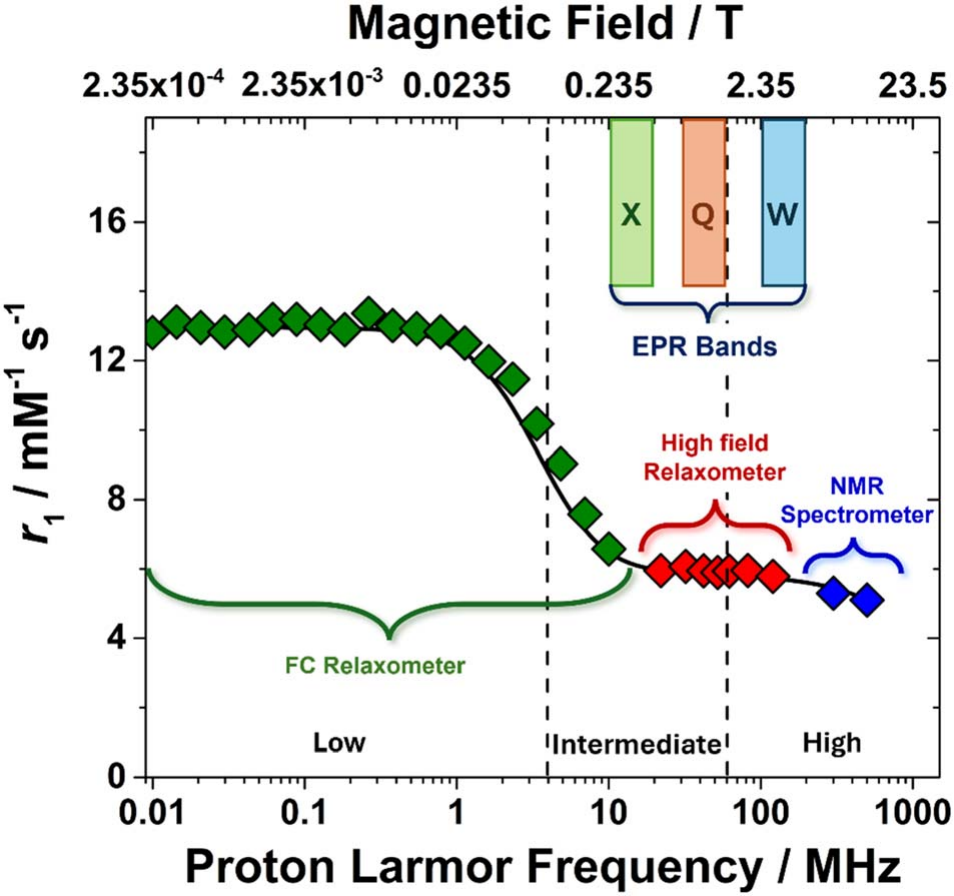
^1^H *T*_1_ NMRD profile of a low molecular paramagnetic chelate highlighting the three characteristic ranges of applied magnetic fields and the corresponding NMR and EPR characterization techniques. The EPR bands correspond to the following frequency-field pairs: X-band (9.5 GHz/0.350 T), Q-band (34 GHz/1.20 T), W-band (95 GHz/3.50 T).

The relaxivity of a paramagnetic complex is not a static property; rather, it varies significantly with the strength of the applied magnetic field. This variation, often expressed as a function of the proton Larmor frequency (*ω*_I_), reflects how efficiently the complex enhances the relaxation rate of water protons across different field strengths. This field dependence is of critical importance because both clinical MRI systems (typically operating at 1.5 T, 3 T, or 7 T) and high-field NMR spectrometers operate within specific magnetic field regimes. As such, the performance of a contrast agent or a relaxation enhancer must be evaluated at relevant magnetic field strengths to ensure optimal effectiveness in the intended application. A contrast agent that performs well at low magnetic field (or low frequency) may become significantly less effective at higher fields due to changes in electron relaxation times and the frequency dependence of *T*_iM_ (proton relaxation time of bound water, eqn (S3)–(S6)). We can identify three typical ranges of applied magnetic field strength (Fig. 4).

Low magnetic fields range (<0.1 T): the relaxivity is often dominated by the electronic relaxation times (*T*_1,2e_) of the paramagnetic metal ion. The dipolar interaction term in the Solomon–Bloembergen–Morgan (SBM) theory is sensitive to *ω*_S_. In some instances, a scalar contribution is observed in this region of the Nuclear Magnetic Relaxation Dispersion (NMRD) profile.

Intermediate magnetic field range (0.1–1.5 T): this is often the most dynamic and informative range, where significant changes in relaxivity are observed. For small to medium-sized complexes, the tumbling of the molecule (*τ*_R_) is a key modulator of the dipolar interaction. Notably this is also the typical magnetic field regime accessible with standard EPR spectrometers: 0.350 T (∼9.5 GHz or X-band), 1.20 T (∼34 GHz or Q-band), 3.50 T (∼95 GHz or W-band).

High magnetic fields range (>1.5 T): as the magnetic field strength increases to very high values, the relaxivity exhibits an additional, characteristic decrease. While the rotational correlation time and the water exchange lifetime remain independent of the magnetic field, the electron relaxation rate generally increases with field strength. However, according to the SBM equation (eqn (S4)), when the proton Larmor frequency reaches very high values (hundreds of MHz), the relaxivity displays a marked decrease (SI). This reduction becomes particularly pronounced for complexes of medium to high molecular weight (*τ*_R_ > 0.5 ns), where molecular reorientation is relatively slow.

The measurement and analysis of this magnetic field dependence are performed through NMRD profiles. Experimentally, this is achieved using a time-domain NMR instrument known as field-cycling (FC) relaxometer,^52,53^ the primary tool for acquiring detailed NMRD profiles. An FC relaxometer is capable of rapidly varying the magnetic field strength over a broad range, typically from a few kHz up to several hundred MHz. The experimental protocol involves three main steps: polarization of the sample at high magnetic field, rapid switching to a lower, precisely controlled “relaxation field” where *T*_1_ relaxation occurs and is measured, and return to high field for signal detection with enhanced sensitivity. This method enables the continuous measurement of *R*_1_ (and in some cases *R*_2_), as a function of the magnetic field, offering critical insight into molecular dynamics and relaxation mechanisms. Data points at higher magnetic fields (*e.g.* >120 MHz) can be acquired using conventional high-field NMR spectrometers. These measurements complement those obtained from FC relaxometers, extending the NMRD profile into the high-frequency range.

The vast majority of available relaxivity data have been obtained for Gd(iii) and Mn(ii) complexes^5,24^ with a growing number of studies in recent years focusing on Fe(iii) complexes.^54–56^ In principle, NMRD profiles depend on numerous structural (*e.g.*, *r*_Me–H_, ^H^*a*_iso_, *q*) and dynamic parameters (*e.g.*, *D*_H_, *τ*_R_, *τ*_M_). Consequently, fitting these profiles using too many free parameters can easily lead to multiple plausible solutions.

To address this, it is standard practice to fix certain parameters at reasonable values, either estimated or obtained from DFT calculations or independent experimental techniques. We will show in the following how EPR spectroscopy can provide detailed measurement of many of such parameters, which combined with advanced DFT methods enable reliable fitting and thorough understanding of the NMRD profiles.

## 3. The EPR perspective

EPR spectroscopy detects species with unpaired electrons and yields detailed information on both their geometric and electronic structure and dynamics. It is therefore the technique of choice to directly answer the key questions related to the role of the electron spin in determining the relaxivity.

EPR shares the same fundamental principles than NMR spectroscopy that is both EPR and NMR probe the interaction of magnetic dipoles with an applied magnetic field and electromagnetic radiation of the appropriate wavelength. Whilst NMR is concerned with the splitting of nuclear spin states in a magnetic field, EPR is concerned with the splitting of electronic spin states. The main differences are: (i) that the magnetic moment of the electron is much larger than any nuclear magnetic moment (at least *ca.* 660 times), as a consequence the energy scale is *ca.* 1000 times larger and the characteristic time scale is also 1000 times faster; and (ii) given the delocalized nature of an electron in the semi-occupied molecular orbital (SOMO) the magnetic moment of the electron cannot be treated as a point dipole and its spatial average must be taken into account.

Unpaired electrons experience a number of magnetic interactions when confined in a molecular system, which impact the energy of the system as quantified by the spin-Hamiltonian. Of these the most relevant for this discussion are the Zeeman and hyperfine interactions, quantified by the **g**- and **A**-matrices. If more than one unpaired electron is present the zero-field splitting matrix must also be considered. The **g**-matrix provides information about the local symmetry of the paramagnet while the **A**-matrix reflects the spin density distribution in the SOMO and the spatial relations between the electron magnetic moment and any nuclear magnetic moment nearby.

Local symmetry, spin density, geometry and dynamics have all direct impact on PRE. Therefore, EPR provides direct chemical and physical information that help the understanding and rationalization of relaxivity, and can guide the rational design of improved contrast agents.

### 3.1 Rotational correlation time

All magnetic interactions are anisotropic. Anisotropies are motionally averaged in fluid solution when the characteristic time scale of the motion exceeds the spectroscopic timescale. However, residual anisotropic contributions are often observed in fluid solution spectra of TMI complexes which can be exploited to derive molecular dynamic parameters. EPR is well suited for this task as the characteristic timescale depends on the inverse of the electron Larmor frequency (*ca.* 0.1 ns at 350 mT), which matches with the characteristic times of molecular motions.

In fluid solution, molecules move according to a Brownian rotational diffusion motion and are said to tumble. The time scale of this motion can be characterized by a rotational correlation time *τ*_R_, which is defined as the time required for a paramagnet to tumble through an arc of 1 rad. *τ*_R_ is related to the rotational diffusion rate constant *D* by *τ*_R_ = 1/6*D*.

For small molecules in non-viscous solvents *τ*_R_ is of the order of 10 ps whereas it reaches values of the order of 1 ns to 100 ns for larger systems, such as proteins and other macromolecules. If the molecule and its solvation sphere can be approximated with a sphere of radius *r* (*i.e.*, *r* is the hydrodynamic radius of the “spherical” solute) in a solvent with viscosity *η*, the rotational correlation time can be estimated by the Stokes–Einstein equation (eqn (3)):3
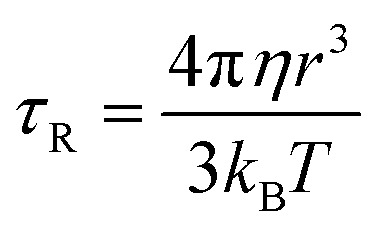


**Fig. 5:**
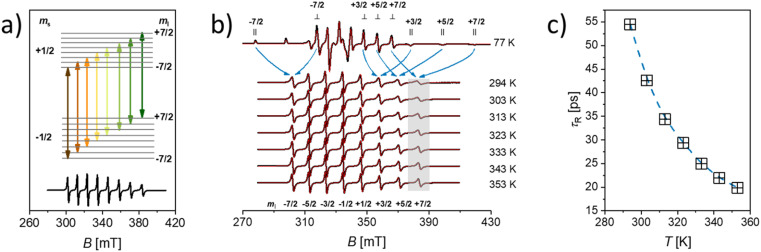
(a) Schematic representation of the energy level diagram of an *S* = 1/2, *I* = 7/2 system in magnetic field with an *a*_iso_ < 0 as it is the case for [VO(H_2_O)_5_]^2+^. The arrows indicate the allowed EPR transitions (Δ*m*_s_ = ±1, Δ*m*_I_ = 0). The [VO(H_2_O)_5_]^2+^ isotropic EPR spectrum is shown as a reference. (b) frozen (77 K) and fluid solution (294 K–353 K) EPR spectra of [VO(H_2_O)_5_]^2+^ (black experimental, red simulated). The arrows indicate the hyperfine lines averaged by molecular tumbling. The shaded area highlights the effect of temperature on the linewidth of the *m*_I_ = +7/2 transition. (c) Temperature dependence of *τ*_*R*_ as extracted from the simulations in (b). Data taken from ref. 25.

To accurately determine *τ*_R_ from EPR data in solution, prior knowledge of the relevant magnetic anisotropies, **g** and **A**, is essential. These can be obtained from solid-state EPR spectra in frozen solution. The effects of rapid tumbling (much faster than the EPR timescale) is an averaging of the **g** and **A** tensor components. The spectrum is therefore mainly determined by the averaged values 
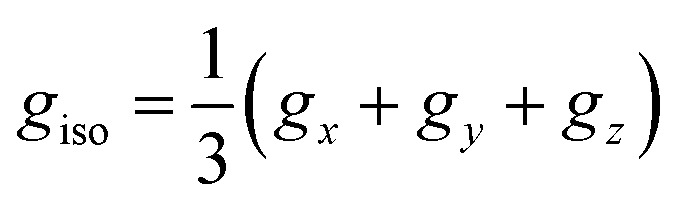
 and 
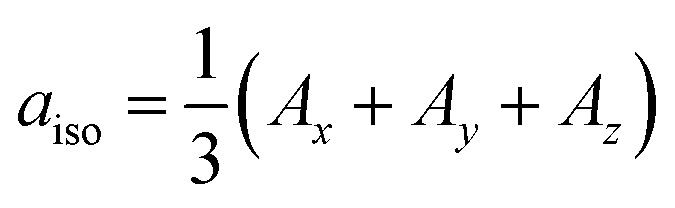
. However, unless the paramagnet is tumbling infinitely rapidly compared to the EPR timescale residual effects of the **g** and **A** anisotropy are still observable and manifest in a broadening of the individual hyperfine lines. A typical example of this is shown in Fig. 5 for the case [VO(H_2_O)_5_]^2+^.

For a paramagnet with *S* = 1/2 the peak-to-peak Lorentzian linewidth can be expressed as a polynomial in the nuclear magnetic quantum number *m*_I_ (eqn (4)):

4 Δ*B*_(p–p)_ = *α* + *βm*_I_ + *γm*_I_^2^ +…

where *α*, *β*, and *γ* are constants containing the rate of tumbling (*τ*_R_) and the anisotropies of **g** and **A**.^57^ Therefore, Δ*B*_(p–p)_ of each EPR line depends on the *m*_I_ value involved in the transition while the second term in eqn (4) imposes a progressive change in the linewidth across the spectrum with the biggest positive *m*_I_ value experiencing the largest broadening. A qualitative way to understand the *m*_I_ dependence of linewidths in fluid solution is to consider how molecular tumbling averages the parallel (‖) and perpendicular (⊥) components of a specific *m*_I_, as illustrated by the arrows in Fig. 5b. The larger the difference in magnetic field between the parallel and perpendicular components being averaged, the faster must be the tumbling to fully average the anisotropy. Consequently, in fluid solution, the resonances associated with a particular *m*_I_ value that exhibit the widest spread are less effectively averaged, resulting in broader lines.^58^ At a given temperature, *τ*_R_ is extracted by fitting the *m*_I_ dependent linewidth of the solution EPR spectrum either phenomenologically through eqn (4) or by solving the stochastic Liouville equation.^59–63^

A more robust estimation involves measuring EPR spectra over a temperature range, enabling the averaging of the **g**- and **A** anisotropies based on the temperature-dependent rate of motion of the paramagnet. Assuming an Arrhenius-type behaviour, this approach also yields the activation energy for the rotational diffusion (Fig. 5c).^25^ Importantly EPR derived *τ*_R_, reflects the tumbling of the entire paramagnetic complex, including its hydration shell, whereas NMR relaxivity specifically depends on the reorientation of the Me⋯H vector (where H is a coordinated water proton). According to SBM *τ*_R_ has an influence on the relaxation time of these protons, *T*_1m_, and therefore affects relaxivity. Generally, slower molecular tumbling (larger *τ*_R_) leads to enhanced relaxivity. For approved contrast agents *τ*_R_ is of the order of 0.1 ns, increasing it, *e.g.* by conjugating the complex to bulky carriers like dendrimers, polymers, or proteins, has proven effective in improving relaxivity.^26^

A few words of caution are appropriate at this stage. In fact, the EPR approach described here requires (1) that an EPR spectrum is observed at room temperature with (2) sizable anisotropic interactions. The former usually excludes TMI in high spin states, with the notable exception of Mn^2+^ (*S* = 5/2). However, for Mn^2+^, the small anisotropies limit the applicability of the approach and may benefit from a multifrequency analysis.^64^

### 3.2 Electron relaxation times

An EPR experiment involves the absorption of electromagnetic radiation by unpaired electrons, resulting in a reversal of the orientation of their magnetic moment in the presence of an external magnetic field. Following this energy absorption, relaxation processes must occur to dissipate the excess energy and return the system to thermal equilibrium.^65–67^ The longitudinal (spin–lattice) relaxation time, *T*_1_, quantifies the rate at which the longitudinal magnetization (aligned along the direction of the static magnetic field *B*_0_*i.e.* along the *z*-axis) returns to its equilibrium value. This occurs through energy transfer from the electron spin system to the surrounding lattice (thermal bath). A second process characterized by the transverse relaxation time (*T*_2_) describes the loss of phase coherence of the transversal magnetization (in the *xy* plane) due to interactions among spins. In the physics literature *T*_2_ is often referred to as the coherence time. In solids *T*_1_ vastly exceeds *T*_2_, however in fluid solution at room temperature the two times constants converge, with *T*_1_ often becoming the limiting factor.

In solution, three main relaxation mechanisms to *T*_1_ can be present: (i) spin rotation; (ii) modulation of **g**- and/or **A**-anisotropy; and iii) modulation of the zero-field splitting (ZFS) for *S* > 1/2. All these processes depend on the magnitude of the corresponding anisotropies which is modulated by the molecular tumbling. In turn, the effectiveness of a particular process is also affected by the working microwave frequency, *i.e.* the larger the microwave frequency the faster the tumbling needed to achieve the same averaging effect.

For a *S* = ½ system, the cumulative effect of all these processes can be summarized as follows (eqn (5)):5
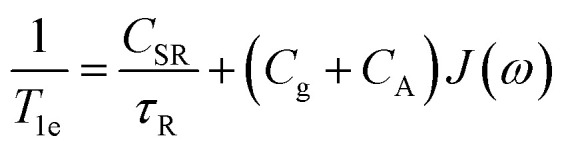
Here *τ*_R_ is the rotational correlation time, *C*_SR_ is the spin rotation coefficient, proportional to (Δ*g*)^2^ (*i.e.* Δ*g* = *g* − *g*_e_, with *g*_e_ = 2.0023) and reflects the spin–orbit contribution to the ground state. *C*_g_ is the coefficient related to **g** anisotropy proportional to the square of the anisotropy and to *ω*_e_^2^ where *ω*_e_ is the electron Larmor frequency. *C*_A_ is the coefficient associated to the modulation of the squared **A** anisotropy. *J*(*ω*_e_) is the spectral density of the paramagnet motion as defined by the Bloembergen–Pound–Purcell model and takes the form (eqn (6)):6
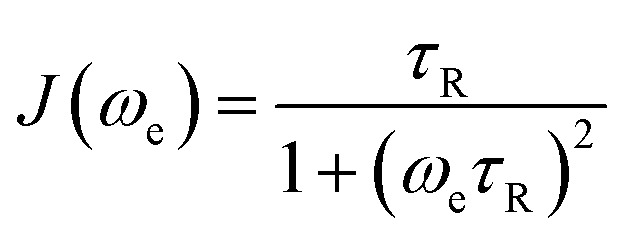


For *S* > 1/2, such as Gd(iii) and Mn(ii), the equations describing electron relaxation in SBM theory need including the ZFS interaction and are reported in the SI (eqn (S13) and (S14)).

In a rigid lattice (or frozen solutions) and neglecting nuclear spins, *T*_2_ is dominated by the orientation-dependent (static) dipolar interaction between the unpaired electrons, therefore *T*_2_ is approximately proportional to 1/*r*_ee_^3^, where *r*_ee_ is the distance between unpaired spins, as shown by Bloembergen *et al.*^46,47^ For a uniform distribution of spins 1/*r*_ee_ = 3√*C* × *N*_A_, *i.e.* the inverse of the average distance depends on the concentration, *C* (expressed in moles per cubic meter), and *N*_A_ is the Avogadro's number.

Measurements of electron relaxation times in fluid solution are typically performed either *via* saturation recovery experiments, in which the EPR signal is observed after the irradiation with a long and weak pump pulse, or *via* saturation curves, in which the EPR signal intensity is monitored as a function of the applied microwave power. While the former can be performed both in CW and pulsed mode, the latter is typically limited to CW measurements and only provides the product of *T*_1_ and *T*_2_. If solid samples are available the most common method is the inversion recovery pulse sequence, which however requires a sufficiently long *T*_2_ to detect an electron spin echo. The experimental determination of electronic relaxation times can be used to access the nuclear relation times as recently shown at low temperature for Gd(iii) complexes^68^ and nitroxide radicals.^69^

### 3.3 Electron spin density distribution and electron nuclear distances

The interaction between the magnetic moment of the unpaired electron and the magnetic moment of a nucleus gives rise to the well-known hyperfine interaction, which leads to the characteristic multiline patterns observed in standard EPR spectra – provided that its magnitude exceeds the intrinsic linewidth (>15–30 MHz for *S* = ½ TMIs). Weaker hyperfine interactions – critical for understanding relaxivity – can be detected using hyperfine spectroscopies. These techniques offer sub-MHz resolution and enable the measurement of the NMR spectra of magnetic nuclei coupled to the electron spin. The main methods include ENDOR (electron nuclear double resonance),^70^ ESEEM (electron spin-echo envelope modulation), HYSCORE (hyperfine sublevel correlation)^71^ and EDNMR (Eldor-Detected NMR).^72^ The isotropic hyperfine coupling, ^H^*a*_iso_, is directly proportional to the electron spin density at the proton, ρ_H_^α−β^, according to (eqn (7)):7
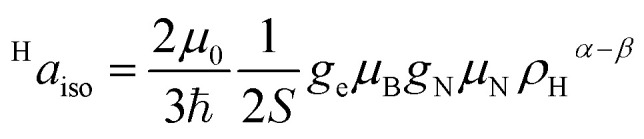
where *μ*_0_ is the vacuum permeability, *g*_e_ is the electron *g*-factor, *μ*_B_ is the Bohr's magneton, *g*_n_ is the nuclear *g* factor and *μ*_N_ is the nuclear magneton, which for the proton have values 5.5857 and 5.05078317(20) × 10^−27^ J T^−1^, respectively.

In the context of contrast agents, this interaction causes the so-called contact shifts, which are a consequence of the delocalization of unpaired electron spin density across chemical bonds. For this reason, they are only observed for nuclear spins fairly close to the paramagnetic center in terms of number of bonds.

Hyperfine spectroscopy not only measures the electron spin density at the proton, but can also provide a direct measure of the electron proton distance through the dipolar interaction between the metal and the nucleus (Table 2). The orientation dependent dipolar interaction, known as anisotropic hyperfine interaction, *T*, in a static point-dipole approximation takes the form:8
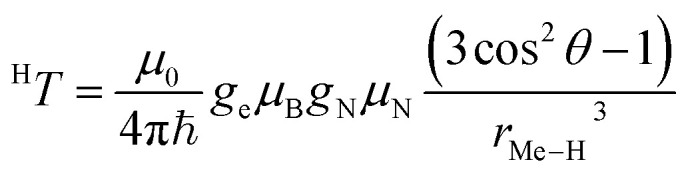
With a good approximation, for protons in transition metal complexes, the spin density can be assumed to be localized at the central metal ion and the typical metal–proton distance (>2.5 Å) justifies the adoption of eqn (8). In this way, the distance between the proton and the central ion, *r*_Me–H_, can be directly derived for a rigid (frozen) system. Frozen samples represent a snapshot of a fluid solution and not only provide the mean distance and spin density at the proton but also the corresponding distribution of orientations and distances. The latter is particularly important, since all contrast agents in solution experience a set of conformations due to, for instance, thermal vibrations and collisions with the solvent. The hyperfine approach on an ensemble allows the quantification of such variability and provide an accurate estimate of two relevant parameters – ^H^*a*_iso_ and *r*_Me–H_ – that impact on relaxivity. Metal–proton distances derived by EPR are therefore far more truthful than data obtained from diffraction methods on single crystals which lack the intrinsic variability present in solution. In combination with proper DFT modelling (*vide infra*) these experimental data yield atomistic structure but, more importantly, structure–properties correlations which can guide the rational design of first-row contrast agents. We note that the metal–proton distance can be obtained by ENDOR or other hyperfine spectroscopies even in the more challenging cases of TMIs and lanthanoids (Gd^3+^) in high spin states.^73,74^ As a final remark, hyperfine spectroscopy, in particular ESEEM, can be used to provide an estimate of the number of coordinated water molequles (*q*).^75^

**Table 2:** Dependence of the accessible electron-nuclear distance (*r*) as a function of the magnetic quantum number (*I* for nuclei, or *S* for the electron) and gyromagnetic ratio (*γ*/2π). In the examples, a dipolar interaction of 1 MHz was assumed. The analogue case for the electron-electron interaction is reported for comparison

	Magnetic quantum number	*γ*/2π (MHz T^−1^)	*r* (Å)
e	1/2	−28024.9514	37.3
^1^H	1/2	42.5774	4.3
^2^H	1	6.5359	0.7
^13^C	1/2	10.7084	1.8
^14^N	1	3.0777	0.3
^17^O	5/2	−5.7742	0.6
^19^F	1/2	40.0776	4.0
^31^P	1/2	17.2514	1.7

### 3.4 ^17^O EPR, NMR and water exchange kinetics

The synergistic use of ^17^O EPR and NMR is a powerful tool to study of the nature of the chemical bond between the paramagnetic metal and the oxygen atom of water binding molecules and a unique source of information concerning dynamic processes. ^17^O is the only magnetic isotope of oxygen. It is characterized by a high spin quantum number (*I* = 5/2) and very low natural abundance (0.038%). This implies that isotopic enrichment is necessary for both EPR and NMR studies as shown by Merbach *et al.* for Gd^3+^ chelates.^76^

From ^17^O hyperfine data, typically obtained through the use of hyperfine techniques, the spin density at the oxygen can easily be estimated. For instance, for the simple case of an unpaired electron (free electron, *g*_e_ = 2.0023) on an s-type orbital of a ^17^O nucleus with a unitary spin population (*ρ*_s_ = 1) the observed isotropic hyperfine coupling constant is ^O^*a*_0_ = −4622.83 MHz. If the electron resides in a p-type orbital, the observed axial hyperfine constant is ^O^*b*_0_ = 130.5 MHz. Including a correction for the difference in the *g* values, the spin populations in s-type and p-type orbitals can thus be estimated as (eqn (9) and (10)):9
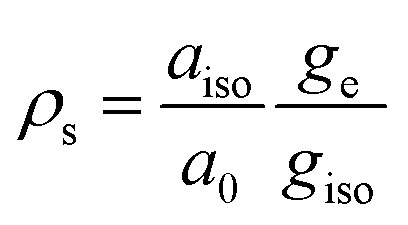
10
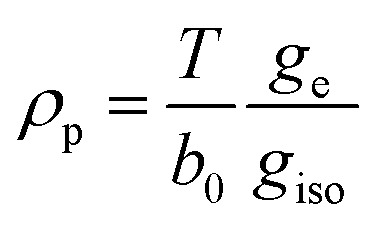


These values are extremely sensitive on the nature of the chemical bonding, allowing to discriminate between s and p interactions, as well as assessing the degree of covalency in the chemical bond.^77^

On the other hand, ^17^O NMR spectroscopy proves to be a powerful tool for probing the dynamics of water exchange in paramagnetic metal complexes. By enriching water with the ^17^O isotope, we can monitor the transverse relaxation rate (*R*_2p_) and chemical shift (Δ*ω*_p_) of the water signal as it interacts with the metal centre. By systematically measuring *R*_2p_ and (Δ*ω*_p_) of the bulk water's ^17^O NMR signal as a function of temperature, the water exchange rate (*k*_ex_) can be accurately extracted.^78^ This method works because the observed relaxation and chemical shift are weighted averages of the inner-sphere and bulk water properties, with the exchange rate dictating how effectively the rapid relaxation of the bound water is communicated to the bulk.

## 4. Computational methods

Theoretical methods have evolved in terms of methodologies, algorithms and software to a point where they have become accessible to a wide community of chemists.^90^ These developments have made theoretical chemistry a key tool in the design and understanding of metal-based MRI contrast agents by enabling the prediction of structural, electronic, and dynamic properties. The introduction of density functional theory (DFT), along with the ongoing development of more accurate functionals, has opened the door to modelling metal complexes with unprecedented accuracy at a reasonable computational cost.^91^ Moreover, *ab initio* methods such as the complete active space self-consistent field (CASSCF) and other multireference approaches can now be applied to relatively large complexes, allowing for the accurate calculation of key properties that are often beyond the scope of DFT.^92,93^ Molecular dynamics, either classical or *ab initio*, can also be applied to metal complexes in aqueous environments, providing access to key dynamic properties occurring in the ps and even ns timescales (*i.e.* rotational and translational diffusion, water exchange rates).^94,95^

For example, the number of coordinated water molecules (*q*) can be determined using both quantum mechanical and molecular dynamics (MD) simulations.

Special care must be taken when applying DFT methods to model MRI contrast agents in order to obtain reliable results. First, the conformational space must be thoroughly explored to ensure that the structure being modelled corresponds to a global minimum on the potential energy surface. Second, solvent effects must be appropriately accounted for. The use of polarizable continuum models (PCM) can enhance the accuracy of computed structures; however, these models have limitations – particularly when the ligand contains negatively charged groups such as carboxylates or phosphonates. In such cases, the inclusion of a few explicit water molecules is often necessary to achieve accurate structural parameters.^96^*Ab initio* MD simulations (*i.e.* Car–Parrinello) can be performed, but their computational cost is high, and thus only relatively short simulation times are typically accessible.^97^ In the case of lanthanide complexes, the use of metadynamics allowed to access activation energies occurring in the ns and even μs timescales, such as water and proton exchange.^98^

Particular attention is needed when DFT is used to target EPR parameters (hyperfine coupling constants and **g** tensor). In this context a great deal of work has been done to evaluate the sensitivity of computational predictions with respect to the functional, basis set, and the different frameworks to account for relativistic effects. Important observations have been made concerning the capabilities and limitations of different approaches in capturing the essential physics, leading to practical suggestions such as the requirement for Hartree–Fock exchange admixture.^99–103^ Coupled cluster calculations including singles and doubles excitations (*i.e.* the domain-based local pair natural orbital approach, DLPNO-CCSD) was also used for the prediction of **A**-and **g** tensors.^104^

Theoretical methods can also be employed to investigate electron spin relaxation, a key parameter for understanding the relaxation properties of contrast agents. *Ab initio* spin dynamics simulations based on open quantum systems theory, DFT and multiconfigurational quantum chemistry techniques are nowadays considered a gold standard tool to perform spin relaxation simulations.^105^ These approaches have been successfully applied to a wide range of magnetic systems, including spin-1/2 species^106–108^ and single-molecule magnets.^109–112^ Recently, an accelerated computational framework based on machine-learning models for the prediction of molecular vibrations and spin–phonon coupling coefficients has been proposed.^113^ This approach is expected to significantly broaden the applicability of numerical simulations for studying spin relaxation, including in the context of MRI contrast agent candidates – an area where such methods have yet to be fully explored.

## 5. Case study

Most of the TMI complexes investigated as MRI contrast agent candidates to date are based on Mn^2+^ and Fe^3+^ complexes with polyamino polycarboxylate ligands (Fig. 1), as well as Mn^3+^ porphyrins.^24,56,114,115^ However, the amount of available data for these complexes is significantly less extensive and far less systematic compared to that for Gd^3+^ complexes. For Gd^3+^ complexes, a wealth of experimental and computational data, accumulated over more than 35 years and spanning hundreds of individual complexes, has led to well-established procedures for assessing the value of several key parameters. This extensive dataset allows researchers to either precisely determine the value of individual parameters or estimate their value within a narrow range, consistently demonstrating their weak dependency on the specific ligand structure.^50,85,116–124^

For instance, the vast number of recorded and analyzed ^1^H NMRD profiles, along with available X-ray crystal structures and ENDOR studies, have consistently demonstrated that the Gd–H distance varies within a narrow range of 3.1 ± 0.1 Å. The rotational correlation time (*τ*_R_^298^) can be accurately estimated using empirical relationships between relaxivity and molecular weight (for a given hydration number *q*). Conversely, the hydration number itself can be extracted from luminescence data obtained from isostructural Eu(iii) and/or Tb(iii) complexes. In addition, ^17^O NMR data can be routinely used to investigate water exchange kinetics once *q* is known, as the hyperfine coupling constant (^O^*a*_iso_) also exhibits a negligible dependence on the nature of the co-ligands.

The analysis of relaxation data in TMI complexes is considerably more complex. For example, methods for determining the hydration number have been developed almost exclusively for Mn^2+^ complexes and are not readily applicable to other TMIs.^125,126^

Moreover, it is generally more difficult to identify isostructural surrogates for TMIs as compared to Ln(iii) ions,^127,128^ making the use of integrated, multi-technique approaches essential. This challenge is particularly acute for ions such as VO^2+^ and Cu^2+^,^25,129,130^ for which relaxometric studies remain scarce, despite their recently demonstrated promising magnetic and coordination properties.^26^

**Fig. 6:**
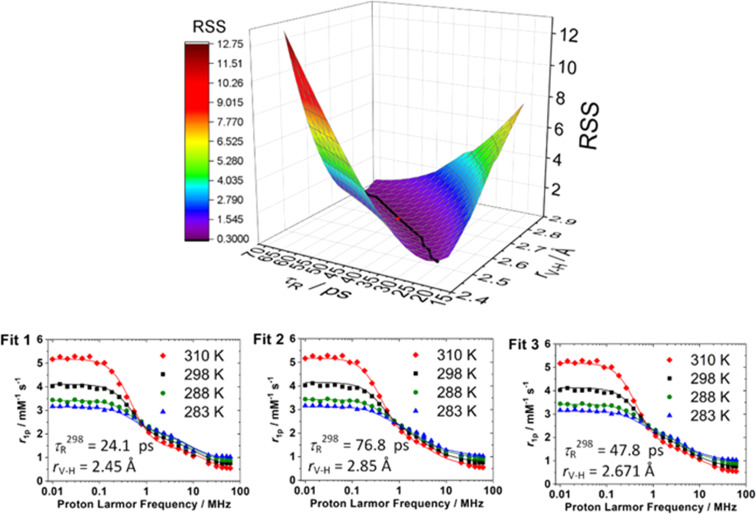
^1^H NMRD profiles recorded for the [VO(H_2_O)_5_]^2+^ showing three different fits (see text) and dependence of the residual sums of squares (RSS) of the fit on *r*_V–H_ and *τ*_R_^298^. The black line on the surface represents the approximate path that minimizes RSS and the red spot signals the results of Fit 3.

To illustrate this, Fig. 6 presents ^1^H NMRD profiles of the [VO(H_2_O)_5_]^2+^ complex, recorded at four different temperatures. This complex was recently characterized by some of us using a comprehensive approach that combined EPR spectroscopy, NMRD measurements, and computational modeling.^25^ It is evident that the NMRD profiles exhibit two distinct dispersion regions: a broad feature spanning approximately 2 to 40 MHz, and a more pronounced dispersion in the low-field region between 0.1 and 1 MHz. The presence of this low-field dispersion strongly suggests a significant scalar (Fermi contact) contribution to the overall relaxivity.

The NMRD profiles were fitted using the Solomon–Bloembergen–Morgan equations, which involves nine independent parameters, several of which exhibit strong interdependence. In particular, the dipolar relaxation contribution depends critically on *q*, *r*_V–H_ and *τ*_R_. As a result, a reliable analysis of the NMRD data requires independent knowledge of at least two of these parameters to avoid overparameterization and ensure meaningful fitting. As illustrated in Fig. 6, equally satisfactory fits can be obtained using different combinations of *r*_V–H_ and *τ*_R_^298^, highlighting the strong correlation between these two parameters in the fitting model. For example, fitting the NMRD data of [VO(H_2_O)_5_]^2+^ using the DFT-derived *r*_V–H_ value of 2.671 Å yields a *τ*_R_^298^ value that is in excellent agreement with the experimental value obtained from EPR (see Fit 3, Table 4). To further assess the reliability of the fits, Fig. 6 shows the residual sum of squares (RSS) associated with different parameter sets, providing a quantitative evaluation of the goodness-of-fit and illustrating the sensitivity of the model to specific parameter choices. The plot of the RSS values, obtained from fitting the NMRD data while varying both *r*_V–H_ and *τ*_R_^298^, reveals a broad and shallow minimum. Specifically, equally acceptable fits can be achieved across a wide range of parameters: *r*_V–H_ values between 2.46 and 2.86 Å and *τ*_R_^298^ values between 14.8 and 74.8 ps. This indicates that multiple parameter combinations yield similarly good fits, due to a compensatory relationship between *τ*_R_^298^ and *r*_V–H_: a shorter rotational correlation time can be offset by a shorter electron–proton distance, and *vice versa* (Fits 1 and 2, respectively). Because of this strong correlation, it is essential to constrain at least one of these parameters using independent experimental information. Such complementary data greatly improves the reliability and confidence of the fit and ensures physically meaningful parameter estimation. This is illustrated in Table 4, which shows that the RSS value decreases significantly when both *τ*_R_^298^ and *r*_V–H_ are fixed to the values obtained from EPR and DFT, respectively. Furthermore, the RSS value obtained and the fitting parameters fall very well on the pathway that minimises RSS for combinations of *τ*_R_^298^ and *r*_V–H_ (Fig. 6).

This strategy not only reduces the effective parameter space but also provides an opportunity to validate DFT-calculated models (see Section 4), which offer atomistic and electronic-level insight into the structure of the complex. A particularly relevant case is the hyperfine coupling constant between the vanadium centre and coordinated water protons, summarized in Table 4, which serves as a critical benchmark for both computational and spectroscopic validation.

As discussed in Section 3.3, the hyperfine tensor provides two key pieces of complementary information: the electron spin density at the proton and its distance to the metal centre. Both parameters can be determined *via* ENDOR spectroscopy, which is performed in the solid state at cryogenic temperatures. In the case of the [VO(H_2_O)_5_]^2+^ complex, ENDOR is capable of resolving the two inequivalent protons on each of the four equatorially coordinated water molecules (see Table 3). By contrast, NMRD measurements are carried out in fluid solution, where dynamic averaging renders all protons magnetically equivalent. The average *r*_V–H_ value obtained from analysis of the dipolar hyperfine tensor and the average isotropic hyperfine coupling constant ^H^*a*_iso_ (Table 3) are therefore the most accurate experimental constraints to improve the accuracy and reliability of the fitting of the relaxivity. While Fig. 6 illustrates the correlation between a specific pair of fitting parameters, similar correlation plots can be generated for all parameter pairs listed in Table 4, providing a comprehensive view of the sensitivity of the model and the robustness of the fitting procedure.

**Table 3:** Selected examples of hyperfine and structural parameters for hydrated paramagnetic metal ions. The dominant contribution of the metal orbital to the SOMO is reported only for *S* = 1/2 3d TMI ions. H_1_ and H_2_ denote the inequivalent protons of water, whereas H_a_ (O_a_) and H_e_ (O_e_) label axial and equatorial nuclei of the same water molecule. All hyperfine couplings are reported in unit of MHz, while the *r*_Me–H(O)_ distances are in Å

	Ti^3+^	VO^2+^	Mn^2+^	Cu^2+^	Gd^3+^
S	1/2	1/2	5/2	1/2	7/2
SOMO	d_*z*^2^_	d_*xy*_	—	d_*x*^2^ − *y*^2^_	—
^H^ *a*_iso_	4.8 (H_1_)	7.7–8.7 (H_1_)	1.0 (H_a_)	0.87 (H_a_)	0.03
	7.5 (H_2_)	−0.05–4.6 (H_2_)	1.0 (H_e_)	1.3 (H_e_)	
*r*_Me–H_ (EPR)	2.7 (H_1_)	2.5 (H_1_)	2.8	2.84	3.09
	2.9 (H_2_)	2.9 (H_2_)		2.90	
^O^ *a*_iso_	7.5	5.0 (O_a_)	7.5	<2 (O_a_)	0.75
		7.16 (O_e_)		50 (O_e_)	
		8.4 (=O)			
*r*_Me–O_	2.097 (DFT)	2.05 (O_a_)	2.18	2.30 (O_a_)	2.37 sol
		2.04 (O_e_)		1.97 (O_e_)	2.346–2.460
Ref.	79	80	82	81	85
		81	83	82	86
		82		83	87
		83		84	88
					89

**Table 4:** Selected parameters obtained from the fits of ^1^H NMRD data of [VO(H_2_O)_5_]^2+^ and residual sums of squares (RSS) values^a^

	Fit 1 (NMRD)	Fit 2 (NMRD)	Fit 3 (NMRD/DFT/EPR)
*τ*_R_^298^/ps	24.1 ± 0.7	76.8 ± 2.2	47.8^c^
*T*_1e_^298^/ns	1.7 ± 0.9	0.95 ± 0.09	1.18 ± 0.11
^H^ *a*_iso_/MHz	5.19 ± 1.5	3.30 ± 0.26	3.97 ± 0.32
*r*_V–H_/Å	2.45^b^	2.85^b^	2.671^d^
RSS	0.662	0.605	0.323

a The diffusion coefficient was set to *D*_V–H_^298^ = 20.3 × 10^−10^ m^2^ s^−1^ and its activation energy was constrained to *E*_D_ = 17.0 kJ mol^−1^, ref. 131.

b Fixed to arbitrary value.

c Fixed to the EPR value.

d Fixed to the DFT value.

In summary, this example clearly demonstrates that EPR data not only provide experimental access to key parameters governing nuclear magnetic relaxation in paramagnetic complexes, but also, when integrated with ^1^H NMRD measurements and computational results, enable a comprehensive, accurate, and unambiguous analysis of relaxometric data. This integrated approach significantly enhances the reliability and interpretive power of the relaxation study.

## 6. Outlook

The development of MRI contrast agents based on first-row TMIs represents a promising strategy to address the safety, environmental, and supply chain limitations associated with gadolinium-based agents. Research on Mn(ii) complexes is well-established, with a growing interest in investigating Fe(iii)-based systems. However, complexes of other metal ions also exhibit interesting and potentially promising properties. For instance, V(iv) complexes have recently been explored and have shown some encouraging features for their use as MRI probes.^25^ Some complexes of paramagnetic TMIs exhibit a strong dependence of *R*_1_ on the magnetic field, a property that can be exploited by fast field-cycling (FFC) MRI to generate image contrast based on the slope d*R*_1_/d*B*_0_ of the agent's NMRD profile.^132^ Significantly, certain V(iv) complexes show a notable relaxation dispersion at low magnetic fields, a property that could be specifically leveraged for this purpose. Alternatively, they could be used as CAs at a fixed low magnetic field (0.25–1 T), as low-field MRI is experiencing a renascence due to improvements in technology.^133^ Although up to now it has largely been overlooked as a candidate for MRI contrast agents, progress made on the use of Cu(ii)^24^ has shown that a Cu(ii) site coordinated exclusively by oxygen donor atoms – belonging to a protein scaffold – exhibits relaxivity values significantly higher than expected and comparable to those of Gd(iii) complexes.

However, realizing this potential demands a paradigm shift in how we understand and design these complexes. Unlike Gd(iii)-based systems – where decades of extensive data and well-established models exist – TMI-based agents pose unique challenges due to their more complex electronic structures, variable coordination geometries, and smaller magnetic moment.

In this perspective, we have outlined a comprehensive, integrative methodology that combines EPR spectroscopy, NMRD measurements, and advanced computational modelling. This approach enables the precise and independent determination of key parameters such as rotational correlation times, electron–proton distances, and electron spin densities – parameters that are often strongly correlated and challenging to extract reliably from NMRD fitting alone. As demonstrated in the case study on [VO(H_2_O)_5_]^2+^, EPR-derived constraints significantly improve the robustness and accuracy of relaxometric analyses, offering deeper insights into structure–property relationships and guiding rational design strategies. EPR spectroscopy, especially when combined with advanced quantum chemical simulations, shifts the common workflow, as it focuses on the electron spin, which is the driver behind relaxation enhancement. By a careful combination of advanced pulsed techniques and temperature-dependent experiments, EPR can provide an independent estimate of many crucial parameters, including average metal–proton distance in solution, electron spin density at the proton and rotational correlation time.

This not only helps to avoid overparameterization – ensuring meaningful fitting of NMRD profiles – but also offers a valuable means to validate DFT-calculated models, thereby bridging experimental and theoretical insights into complex structure–property relationships.

Looking forward, several promising directions emerge. First, the integration of emerging machine-learning frameworks with quantum mechanical models offers opportunities for accelerating the prediction of vibrational and spin–relaxation properties. Second, the largely untapped potential of *S* = ½ systems such as Cu(ii) and VO(iv), particularly as redox-responsive “smart” probes, warrants further systematic exploration. Finally, the development of standardized experimental workflows that leverage the complementarity of EPR, NMR, and theoretical modelling will be essential for building a comprehensive database of TMI-based agents – analogous to what currently exists for Gd(iii) systems. This will facilitate the timely discovery of much needed alternatives.

In conclusion, a concerted multidisciplinary effort that tightly integrates spectroscopy, theory, and relaxometry is not only desirable but necessary to unlock the full potential of first-row transition metals in MRI contrast agent design. This approach will not only enhance diagnostic safety and versatility but may also open up entirely new avenues in functional and responsive molecular imaging.

## Author contributions

Conceptualization (Enrico Salvadori, Mauro Botta, Carlos Platas-Iglesias, Mario Chiesa). Funding acquisition (Enrico Salvadori, Fabio Carniato, Mauro Botta, Carlos Platas-Iglesias, Mario Chiesa). Visualization (Valeria Lagostina, Marco Ricci, Fabio Carniato). Writing – original draft (Enrico Salvadori, Mauro Botta, Carlos Platas-Iglesias, Mario Chiesa). Writing – review & editing (Enrico Salvadori, Valeria Lagostina, Marco Ricci, Fabio Carniato, Mauro Botta, Carlos Platas-Iglesias, Mario Chiesa).

## Conflicts of interest

There are no conflicts to declare.

## Supplementary Material

SC-OLF-D5SC05827A-s001

## Data Availability

No primary research results, software or code have been included and no new data were generated or analysed as part of this review. Supplementary information: details on the inner sphere and outer sphere contributions to relaxivity are provided. See DOI: https://doi.org/10.1039/d5sc05827a.
