# Supplementary material for: From electron spin to relaxivity: a multidisciplinary perspective on first-row transition metal-based MRI probes
Source: Chem Sci. 2025 Oct 20;16(44):20631–46. doi: 10.1039/d5sc05827a (PMC12560143; doi:10.1039/d5sc05827a)
Supplement: SC-016-D5SC05827A-s001 [file SC-016-D5SC05827A-s001.pdf]

Supplementary Information (SI) for Chemical Science.
This journal is © The Royal Society of Chemistry 2025

SUPPLEMENTARY INFORMATION

From Electron Spin to Relaxivity: A Multidisciplinary Perspective on First-Row Transition Metal-Based MRI Probes.

Enrico Salvadori,[a] Valeria Lagostina,[a] Marco Ricci,[b] Fabio Carniato,[b] Mauro Botta [b] Carlos Platas-Iglesias,[c*] Mario Chiesa [a]*

[a] Department of Chemistry, University of Turin, Via Giuria 9, 10125 Torino, Italy.

[b] Dipartimento di Scienze e Innovazione Tecnologica, Università del Piemonte Orientale, Viale T. Michel 11, 15121 Alessandria, Italy

[c] Centro Interdisciplinar de Química e Bioloxía (CICA) and Departamento de Química, Facultade de Ciencias Universidade da Coruña, 15071 A Coruña, Galicia, Spain

* Corresponding authors: mauro.botta@uniupo.it; carlos.platas.iglesias@udc.es; mario.chiesa@unito.it

Table of contents

The inner-sphere (IS) contribution 2

The outer-sphere (OS) contribution 5

References.. 6

The inner-sphere (IS) contribution

Due to the sharp distance-dependent decay of the magnetic field generated by a paramagnetic ion's unpaired electron spins, only nearby water molecules experience significant relaxation enhancement. Among the three contributions, the IS component (r_i^{IS}) is frequently the most significant. This is due to the dominant short-range interactions and because its constituent parameters (e.g., q, τ_M, r_{MH} for inner-sphere water) can often be strategically optimized through ligand design. The r_i^{IS} contribution is mediated by the chemical exchange of protons between the coordinated water molecules and the bulk solvent. For longitudinal relaxation, r_i^{IS} is given by:

$$r_1^{IS} = \frac{Cq}{55.56}\frac{1}{T_{1M} + \tau_M} \qquad \text{(S1)}$$

And for transverse relaxation, r_2^{IS} is given by:

$$r_2^{IS} = \frac{Cq}{55.56}\frac{1}{\tau_M}\frac{T_{2M}^{-1}\left(T_{2M}^{-1} + \tau_M^{-1}\right) + \Delta\omega_M^2}{\left(T_{2M}^{-1} + \tau_M^{-1}\right)^2 + \Delta\omega_M^2} \qquad \text{(S2)}$$

In Equations S1 and S2, C denotes the molar concentration of the paramagnetic ion, T_{1M} and T_{2M} the longitudinal and transverse relaxation times of the protons in the coordinated water molecule(s), and $\Delta\omega_M$ is the chemical shift difference (in rad s^{-1}) between the protons of the metal-bound water and those of the bulk water, measured in the absence of chemical exchange. Note that the term $Cq/55.56$ corresponds to the mole fraction of coordinated (inner sphere) water, P_M. Furthermore, the residence lifetime of the coordinated water molecule plays a crucial role in determining relaxivity. If τ_M is comparable to or longer than T_{1M}, relaxivity becomes limited due to the intermediate exchange regime. In contrast, when the condition $\tau_M << T_{1M}$ is met (fast exchange regime), r_1^{IS} is no longer limited by exchange kinetics but instead depends on q and T_{1M} - that is, on the chemical properties of the metal complex. Conversely, in the slow exchange regime ($\tau_M >> T_{1M}$), r_1^{IS} is significantly reduced but increases with temperature. Therefore, complexes with labile coordination spheres (shorter τ_M, leading to fast or intermediate exchange conditions) are generally more effective at enhancing bulk water proton relaxivity, assuming T_{1M} and T_{2M} are sufficiently short. Furthermore, the relaxation times of the protons in the coordinated water molecule are given (Equation S3) by the sum of the three contributions discussed earlier:

$$\frac{1}{T_{iM}} = \frac{1}{T_{iM}^{DD}} + \frac{1}{T_{iM}^{SC}} + \frac{1}{T_{iM}^{CS}}$$

with *i* = 1,2 (S3)

Each individual contribution, corresponding to a different relaxation mechanism, is described by the following equations - for both longitudinal relaxation (Equations S4-S6) and transverse relaxation (Equations S7-S9).

$$\frac{1}{T_{1M}^{DD}} = \frac{2}{15}\frac{\gamma_I^2 g^2 \mu_B^2}{r_{MH}^6} S(S+1)\left(\frac{\mu_0}{4\pi}\right)^2 \left[\frac{7\tau_{c2}}{1+\omega_S^2\tau_{c2}^2} + \frac{3\tau_{c1}}{1+\omega_I^2\tau_{c1}^2}\right]$$

(S4)

$$\frac{1}{T_{1M}^{SC}} = \frac{2S(S+1)}{3}\left(\frac{A}{\hbar}\right)^2 \left(\frac{\tau_{e2}}{1+\omega_S^2\tau_{e2}^2}\right)$$

(S5)

$$\frac{1}{T_{1M}^{CS}} = \frac{2}{5}\left(\frac{\mu_0}{4\pi}\right)^2 \frac{\omega_I^2 \mu_B^4 \mu_{eff}^4}{(3k_B T)^2 r_{MH}^6}\left(\frac{3\tau_{CS}}{1+\omega_I^2\tau_{CS}^2}\right)$$

(S6)

$$\frac{1}{T_{2M}^{DD}} = \frac{1}{15}\frac{\gamma_I^2 g^2 \mu_B^2}{r_{MH}^6} S(S+1)\left(\frac{\mu_0}{4\pi}\right)^2 \left[\frac{13\tau_{c2}}{1+\omega_S^2\tau_{c2}^2} + \frac{3\tau_{c1}}{1+\omega_I^2\tau_{c1}^2} + 4\tau_{c1}\right]$$

(S7)

$$\frac{1}{T_{2M}^{SC}} = \frac{S(S+1)}{3}\left(\frac{A}{\hbar}\right)^2 \left(\frac{\tau_{e2}}{1+\omega_S^2\tau_{e2}^2} + \tau_{1e}\right)$$

(S8)

$$\frac{1}{T_{2M}^{CS}} = \frac{1}{5}\left(\frac{\mu_0}{4\pi}\right)^2 \frac{\omega_I^2 \mu_B^4 \mu_{eff}^4}{(3k_B T)^2 r_{MH}^6}\left(4\tau_{CS} + \frac{3\tau_{CS}}{1+\omega_I^2\tau_{CS}^2}\right)$$

(S9)

The equations feature a set of correlation times (τ) which describe the various ways the electron-nucleus interactions are modulated over time. These primarily include the rotational correlation time (τ_R), the mean residence lifetime of water molecules in the coordination sphere (τ_M), and the electron spin relaxation times (T_{ie}):

$$\frac{1}{\tau_{ci}} = \frac{1}{\tau_R} + \frac{1}{\tau_M} + \frac{1}{T_{ie}} \quad \text{with i= 1,2} \qquad \text{(S10)}$$

$$\frac{1}{\tau_{ei}} = \frac{1}{\tau_M} + \frac{1}{T_{ie}} \quad \text{with i= 1,2} \qquad \text{(S11)}$$

$$\frac{1}{\tau_{CS}} = \frac{1}{\tau_R} + \frac{1}{\tau_M} \qquad \text{(S12)}$$

Electron spin relaxation is highly dependent on the nature of the metal ion. For ions with *S* free ion ground term, such as Gd(III) (*S* = 7/2), Mn(II), or Fe(III) (both *S* = 5/2), relaxation is primarily governed by zero-field splitting (ZFS) interactions. For metal chelates characterized by highly symmetrical coordination cages static ZFS effects are largely cancelled and only transient ZFS remains, arising from dynamic distortions of the coordination environment. Collisions with solvent molecules can induce the transient distortions in the coordination polyhedron, generating a temporary ZFS. As a result, even in systems with high symmetry that lack static ZFS, spin relaxation can still occur due to momentary symmetry breaking caused by solvent-induced deformations. Under these conditions, electron spin relaxation can be described by the approximate expressions derived by Bloembergen and Morgan (Equations S13-S14):

$$\left(\frac{1}{T_{1e}}\right)^{ZFS} = \frac{2}{50}(4S(S+1)-3)\Delta^2\left(\frac{\tau_v}{1+\omega_S^2\tau_v^2} + \frac{4\tau_v}{1+4\omega_S^2\tau_v^2}\right)$$

(S13)

$$\left(\frac{1}{T_{2e}}\right)^{ZFS} = \frac{1}{50}(4S(S+1)-3)\Delta^2\left(3\tau_v + \frac{5\tau_v}{1+\omega_S^2\tau_v^2} + \frac{2\tau_v}{1+4\omega_S^2\tau_v^2}\right)$$

(S14)

In Equations S13-S14 Δ^2 represents the mean-square ZFS energy and τ_V is the correlation time for the modulation of the zero-field-splitting interaction.

Equations S13 and S14 were originally derived under the assumption that ZFS is modulated by transient, fast distortions of the coordination environment. Subsequent studies, however, have shown that the static component of the ZFS - modulated by the random rotational motion of the complex - must also be taken into account.[1] Nevertheless, the SBM equations can be used to describe electron relaxation assuming that Δ and τ_v represent the effective rotational-vibrational ZFS amplitude and correlation time, respectively.[2,3] In certain cases, such as Mn(III) porphyrins (*S* = 2), unusually large or complex ZFS interactions make the interpretation of relaxometric data within the framework of SBM theory challenging. Additional experimental and theoretical studies are therefore needed to achieve a more complete understanding of the underlying electron spin dynamics.[4,5]

The relaxivity of paramagnetic agents is profoundly influenced by electron relaxation mechanisms, particularly at low magnetic fields (below ca. 2 MHz). Although these field strengths fall outside the conventional range for clinical MRI, a renewed focus is being placed on low-field imaging as a compelling alternative to high-field scanner technology. This includes novel applications like fast field cycling MRI, where agents such as Mn(II) chelates can be strategically utilized for generating contrast. The drive towards low-field MRI is further fuelled by its inherent advantages: it can significantly mitigate image distortion, lower specific absorption rates (a safety concern with high fields), substantially reduce equipment and operational costs, and offer superior imaging performance in anatomical areas prone to susceptibility artifacts, such as air-tissue boundaries.

While the same theoretical framework can describe the SS contribution to relaxivity, precisely localizing these water molecules remains a significant challenge, precluding their individual assessment and analysis. Therefore, the SS contribution to r_1 is usually determined indirectly. By first making the most accurate possible estimations of the inner-sphere (IS) and outer-sphere (OS) contributions, the residual relaxivity is then assigned to the SS component. This SS contribution is subsequently analysed by postulating reasonable values for the q^{SS}/r^6 ratio, effectively treating it as the equivalent contribution from q^{SS} water molecules positioned at an average distance r from the metal ion.

The outer-sphere (OS) contribution

In the absence of inner-sphere water molecules, the longitudinal relaxivity (r_1) of a metal complex can be accurately described using an outer sphere relaxation model, as given by Equation S15.

$$r_1^{OS} = \frac{32\pi}{405}\left(\frac{\mu_0}{4\pi}\right)^2 \frac{N_A[M]}{a_H D_H} \gamma_I^2 \gamma_S^2 \hbar^2 S(S+1)\left[3J_1^{OS}(\omega_I) + 7J_2^{OS}(\omega_S)\right] \tag{S15}$$

where D_H represents the relative diffusion coefficient of the paramagnetic metal ion and water molecules, while a_H denotes the distance of closest approach between an outer sphere water molecule and the paramagnetic center. This diffusion-based model, which assumes non-interacting particle motion, has proven effective not only in explaining the relaxivity values of $q = 0$ complexes (i.e., those lacking coordinated water), but also in reliably describing the OS contribution in $q > 0$ complexes of Gd(III), Fe(III), and Mn(II). This contribution, therefore, does not depend on the rotational dynamics of the complex or on τ_M, but rather on the electron relaxation times.

References

1 S. Rast, A. Borel, L. Helm, E. Belorizky, P. H. Fries and A. E. Merbach, J. Am. Chem. Soc., 2001, 123, 2637–2644.

2 E. Belorizky and P. H. Fries, Phys. Chem. Chem. Phys., 2004, 6, 2341–2351.

3 P. Caravan, C. T. Farrar, L. Frullano and R. Uppal, Contrast Media Mol. Imaging, 2009, 4, 89–100.

4 T. Nemeth, A. Pallier, Ç. Çelik, Z. Garda, N. Yoshizawa-Sugata, H. Masai and Y. Yamakoshi, Chem. Biomed. Imaging, 2024, 3, 5–14.

5 N. Schaefle and R. Sharp, J. Phys. Chem. A, 2005, 109, 3267–3275.
